# Structural Basis for Nucleobase Activation by the Adenine DNA Glycosylase MutY

**DOI:** 10.1002/cbic.70414

**Published:** 2026-06-11

**Authors:** L. Peyton Russelburg, Merve Demir, Karina Cedeno, Sheila S. David, Martin P. Horvath

**Affiliations:** ^1^ School of Biological Sciences University of Utah Salt Lake City Utah USA; ^2^ Department of Chemistry Chemistry and Chemical Biology Graduate Program University of California DavisOne Shields AvenueDavis California USA

**Keywords:** base excision repair, biochemistry, biology, catalysis, chemistry, DNA glycosylase, DNA repair, protein evolution, protein structure

## Abstract

The DNA glycosylase MutY excises adenine when mispaired with oxidized guanine (OG). While it is understood that inappropriate adenine excision would be catastrophic, the mechanism by which MutY activity is kept in check and only licensed at OG:A lesions is unknown. To explore the structural basis for nucleobase activation, we tested kinetic and structural consequences following replacement of the catalytic Glu, a signature residue for MutY. E43Q and E43S substitution variants of MutY from *Geobacillus stearothermophilus*, though severely impaired, retained measurable activity. X‐ray crystal structures showed the substrate nucleobase in an *anti* conformation, rotated by 180° from the *syn* conformation seen in previous substrate complexes. Remarkably, the AP product was observed as the *alpha*‐anomer configuration when generated by these Glu‐replacement variants, completely different from the *beta*‐anomer AP product expected for the wild‐type enzyme and seen directly for other cancer‐associated variants. Our results suggest a mechanism for regulating MutY, whereby Glu engagement with the *syn* conformation of the nucleobase licenses a “*go ahead*” state for adenine excision only at OG:A lesions, while Glu dis‐engagement establishes an “*on hold*” state to avoid inappropriate activity elsewhere.

## Graphical abstract

DNA glycosylase MutY engages its adenine nucleobase substrate in a *syn* orientation with a catalytic Glu. X‐ray crystal structures of MutY with Glu replaced suggest that a disengaged “*on hold*” state with *anti* nucleobase orientation may explain how MutY avoids catastrophic activity at inappropriate adenines.

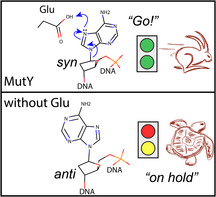

## 1 Introduction

As part of the GO DNA repair system, MutY provides a last line of defense against G:C → T:A mutations that otherwise would result from the oxidatively damaged 8‐oxo‐7,8‐dihydroguanine (OG) lesion [1, 2]. The GO DNA repair system provides multiple, layered responses to OG in DNA and as a free nucleotide and has been reviewed previously [3, 4]. OG is an unreliable template, pairing alternatively with cytosine, *via* normal Watson–Crick–Franklin base pairing, and also with adenine, *via* Hoogsteen base pairing [5]. Ambiguity during DNA replication following oxidative damage generates the pro‐mutagenic OG:A lesion. MutY intercepts OG:A lesions and initiates base excision repair (BER) by cleaving the N–glycosidic bond of the 2′‐deoxy‐adenosine [6, 7, 8]. The importance of intercepting the OG:A mismatch is underscored by the association of inherited MUTYH variants and cancer [9].

**FIGURE 1:**
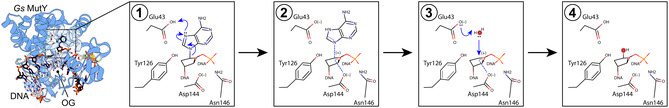
Mechanism of MutY. A glutamic acid (Glu43) initiates glycosidic bond cleavage by protonating N^7^ of adenine (Step 1) and guides stereochemistry of nucleophile attack (Step 3) in the retaining mechanism for MutY. Drawn with Avogadro v1.2.0 [10], on the basis of the structure of the active site as observed in X‐ray crystal structures for the 2′‐fluoro‐2′‐deoxy‐adenine lesion recognition complex (FLRC) (Step 1, PDB ID 3g0q) [11], the TSAC (Step 2 and Step 3, PDB ID 6u7t) [12], and structures of *Gs* MutY (N146S) in complex with the enzyme‐generated AP site (Step 4, PDB ID 8dw3) [13].

The accepted mechanism for adenine excision catalyzed by MutY, shown in Figure 1, features a Glu residue as acid/base catalyst to activate the leaving group (Step 1) and to deprotonate the nucleophile (Step 3). The oxacarbenium ion transition state intermediate (Step 2) results from complete dissociation of the nucleobase leaving group, as determined by kinetic isotope effect (KIE) analysis [14]. This transition state is stabilized by close interaction with a catalytic Asp as revealed by X‐ray crystal structures for the complex of MutY from *Geobacillus stearothermophilus* (*Gs* MutY) with a transition state analog, known as the transition state analog complex (TSAC) [12]. Recent QM/MM calculations show that the Asp residue forms a transient covalent cross‐link with the DNA [15]. The apurinic/apyrimidinic (AP) product retains the *beta* stereoconfiguration as first diagnosed by determination of stereoconfiguration for MutY‐catalyzed methanolysis [12]. Recent X‐ray crystal structures of *Gs* MutY (N146S) complexed with the enzyme‐generated AP site directly demonstrated retention of stereoconfiguration also for the hydrolysis reaction [13].

MutY is tasked with differentiating chemically identical adenines, avoiding most of these, and removing only the rare A paired with OG. MutY will process G:A mispairs in double‐stranded DNA in vitro. However, OG:A is the preferred substrate in vitro and the only substrate processed by MutY in vivo as evidenced by cellular repair assays [1, 16, 17]. To accomplish cross‐strand discrimination, MutY makes extensive contacts with the OG lesion. Residues contributed by the N‐terminal domain (NTD), a Helix‐turn‐Helix domain shared by several BER DNA glycosylases, establish stacking and hydrogen‐bonding interactions that resemble those available for G and OG in Watson–Crick–Franklin base pairs.

Residues contributed by the C‐terminal domain (CTD), a Nudix fold shared with the OG‐nucleotide triphosphatase MutT/MTH, establish contacts only available for OG. Specifically, a serine residue found in a highly conserved FSH motif [18, 19], makes two OG‐specific hydrogen bonds with O^8^ and H^7^, the two atoms present in OG and absent in G [20]. These OG‐specific interactions seen in X‐ray crystal structures are supported by the neighboring Phe and His residues of the FSH motif. Replacement of two or more FSH residues disables mutation suppression function in bacteria [20, 21] and diminishes OG versus G specificity in vitro [20]. SAR studies combined with single‐molecule behavior indicated that the His residue of the FSH motif makes initial contact with an exocyclic amine in the major groove to positively recognize OG while scanning DNA [22, 23, 24]. Although the biochemical and structural basis for OG recognition seems clear, the mechanism by which OG recognition licenses adenine removal is unknown.

Herein, we sought to identify new intermediates that might illuminate the licensing mechanism for MutY. We explored replacement of the catalytic Glu and substitution of adenine with an alternative substrate, purine (dP). *Gs* MutY variants E43S and E43Q were severely impaired yet generated the AP product starting from OG:A and OG:dP, as evidenced by glycosylase assays. X‐ray crystal structures for E43S and E43Q revealed the adenine and purine nucleobases oriented in an unexpected *anti* conformation; all previous structures showed the substrate base in a *syn* like conformation. The enzyme‐generated AP site in complex with E43S *Gs* MutY adopted the *alpha* anomer as diagnosed by 1.68‐Å resolution electron density maps, suggesting a much slower inverting mechanism takes over when the catalytic Glu is unavailable. These results point to a previously unrecognized role for *syn/anti* conformational switching to determine engagement/dis‐engagement with the catalytic Glu.

## 2 Results and Discussion

### 2.1 Glycosylase Activity for E43S and E43Q *Gs* MutY

**FIGURE 2:**
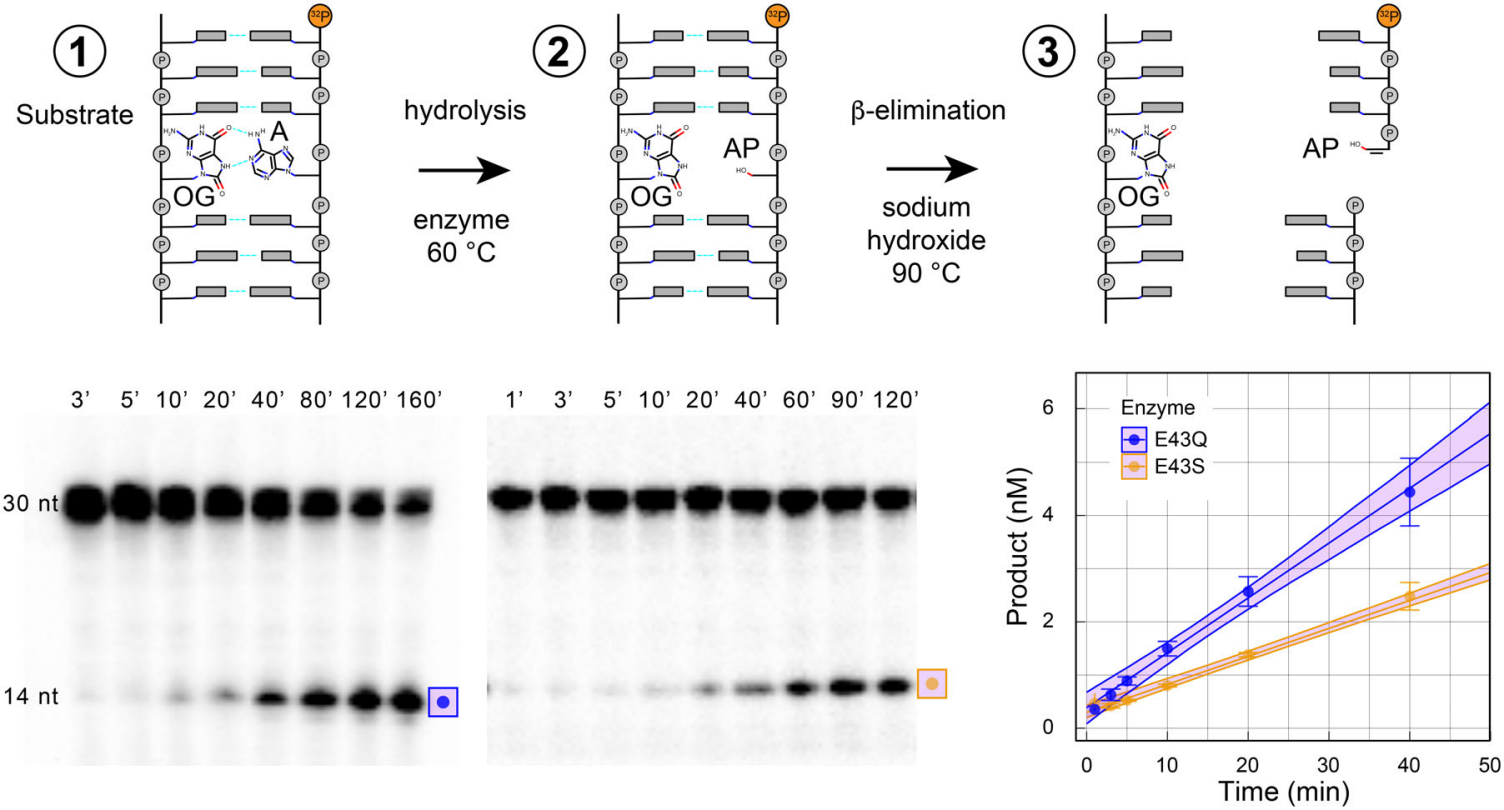
Adenine glycosylase activity for E43Q and E43S *Gs* MutY. DNA (20 nM) containing OG across from adenine with the A‐strand radiolabelled (Step 1, of scheme) was combined with enzyme (1 µM) and incubated at 60°C to yield the AP product (Step 2). Reactions were quenched at specific time points by addition of sodium hydroxide to cleave DNA at the AP site (Step 3). Reactions were analyzed by acrylamide gel electrophoresis to separate bands corresponding with the low‐mobility substrate DNA (30 nt) and high‐mobility product DNA (14 nt). Bands were visualized by autoradiography and quantified. Time course data at early time points with less than 25% product conversion were fit to a linear model to obtain initial velocities. Trials were repeated three times. Error bars represent the sample standard deviation. Shaded regions show the 95% confidence intervals for linear models as determined by bootstrap sampling.

The adenine glycosylase activity of E43S and E43Q *Gs* MutY was monitored under single turnover conditions for a 30 base pair duplex with a central located OG:A mismatch in a manner similar to that previously reported for wild‐type *Gs* MutY [12]. Briefly, the A‐strand was ^32^P‐labeled prior to duplex annealing. The duplex substrate was incubated with E43S or E43Q *Gs* MutY at 60°C and aliquots were removed and quenched at time points over the course of 2 h. Reaction progress was monitored by gel electrophoresis to separate radiolabeled substrate and product DNAs and quantified *via* storage phosphor autoradiography (Figure 2). Fitting of these data, as described previously [6], provided apparent adenine excision rates (*k*_obs_) of 0.0052 ± 0.0008 min^−1^ (95% ci: 0.0044, 0.0059) and 0.0026 ± 0.0002 min^−1^ (95% ci: 0.0024, 0.0028) for the E43Q and E43S enzymes, respectively. Replacement of Glu43 with Gln and Ser drastically slowed the reaction by a factor of 10,000 and 20,000 relative to wild‐type *Gs* MutY, previously characterized with *k*_2_ = 54 (±4) min^−1^ [12].

Replacement of catalytic residues reveals their importance by comparison of the variant and wild‐type enzymes [25, 26, 27, 28]. Our previous work showed that replacement of this catalytic residue in MutY from *E. coli* resulted in a catalytically inactive enzyme that was unable to repair OG:A lesions in a cellular repair assay [29]. In the current work described here, *Gs* MutY enzyme variants with Ser and Gln substituting for the catalytic Glu retained measurable activity. The different outcomes, completely inactive versus severely impaired, likely reflect differences in thermostability. *Gs* MutY is adapted for high temperature, and the assays described in the current work were at 60°C over 2 h, conditions that denature the mesophile enzyme.

For the OG:dP substrate, these enzymes were even slower. In place of precise rate determinations, upper limits for apparent rates were estimated on the basis of observed product accumulation after 3, 6, and 24 h (Figure S1). Product accumulation for enzyme‐catalyzed reactions was greater in comparison to spontaneous background depurination measured without enzyme. For example, after 24 h incubation at 60°C, E43Q converted 25% of substrate to product, and E43S generated 60% product, both significantly greater compared to ∼6% product measured as background for the no‐enzyme control. After subtracting background depurination, we estimated upper limits for enzyme‐catalyzed depurination *k*_obs_ of 3 (±2) × 10^−4^ min^−1^ and 9 (±3) × 10^−4^ min^−1^ for E43Q and E43S enzymes, respectively. In summary, replacement of Glu43 severely impaired catalysis, especially with the purine substrate, yet AP product accumulated to significant levels, an observation that is important to note for the structural analysis.

### 2.2 X‐Ray Crystallography

To better understand mechanistic contributions of the catalytic Glu, we crystallized the E43S and E43Q variants in complex with DNA containing OG paired with adenine (dA) or purine (dP) and determined the structures by crystallography. Diffraction data with synchrotron‐generated X‐rays were measured to high resolution and the structures were determined by molecular replacement. Importantly, the substrate base was omitted from initial refinement so as to observe unbiased discovery maps, which revealed intact substrate for some structures and conversion to the AP (abasic site) product in other cases.

We selected for complete model refinement three representative structures on the basis of data quality (Table 1), and will refer to these structures by the replacing residue at position 43 and the nature of the DNA lesion. For example, E43S_OG:dA_ indicates the E43S variant of *Gs* MutY in complex with DNA containing OG across from adenine. Likewise, E43Q_OG:dP_ denotes the E43Q variant with an OG:purine (OG:dP) lesion, and E43S_OG:AP_ is the structure of E43S *Gs* MutY in complex with the enzyme‐generated apurinic/apyrimidinic (AP) site. In other contexts AP refers generally to any abasic linker within DNA, often the tetrahydrofuran linker, which is a synthetic component not found naturally in DNA. Here, AP signifies the authentic product of the MutY‐catalyzed reaction.

**TABLE 1:** Structures for Glu43 replacement variants of *Gs* MutY.

	E43S_OG:dA_	E43Q_OG:dP_	E43S_OG:AP_
Position 43	Ser	Gln	Ser
DNA active site	Adenine	Purine^a^	AP product
Resolution (Å)	2.60	2.20	1.68
PDB ID	8dwf	8dwe	8dwd

a Partial occupancy, *q* = 0.7.

The substrate and product complexes crystallized in different space groups (Figure S2). After multiple rounds of reciprocal space refinement interleaved with model rebuilding, the final refined models were characterized by high‐quality expectations for *R* values, Ramachandran plots, clash avoidance, and bond geometry (Table S1).

### 2.3 Overview of Structures

**FIGURE 3:**
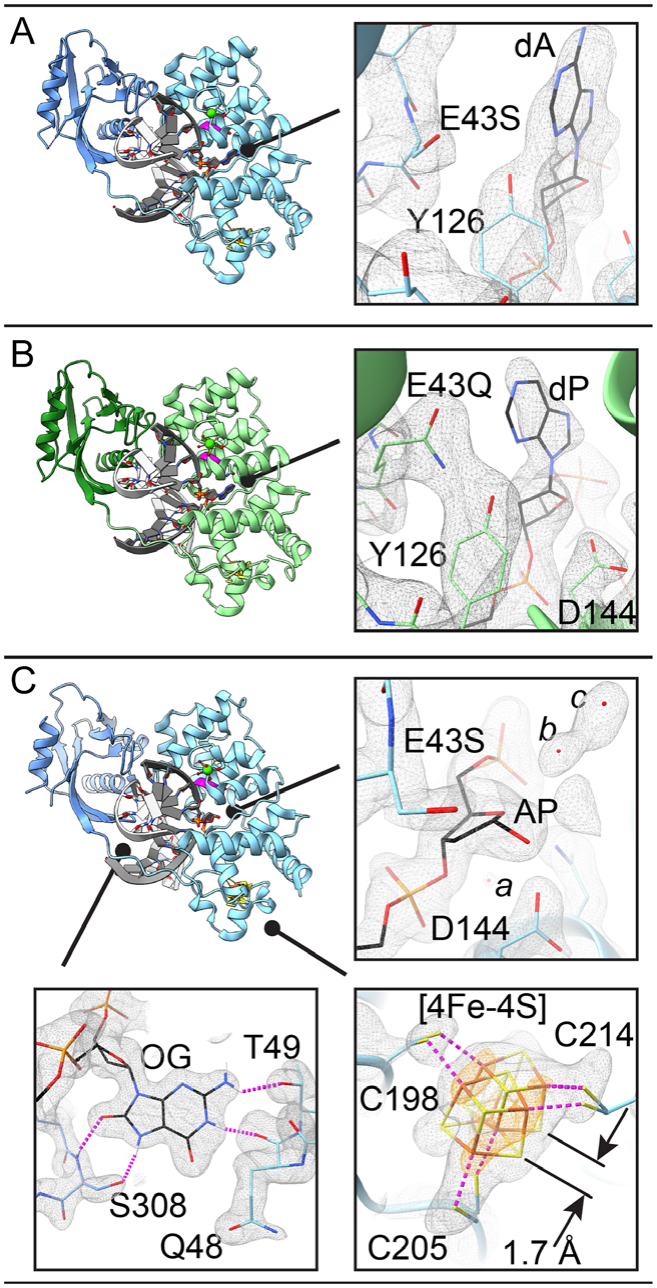
Structures of *Gs* MutY with Glu43 replacement. DNA is colored grey and black. The site of replacement is highlighted with magenta in the ribbon overviews. (A,B) E43S_OG:dA_ (A) and E43Q_OG:dP_ (B) are shown with detailed views of the active site with simulated annealing composite omit maps (grey) contoured at the 1‐*σ* r.m.s.d. level. (C). E43S_OG:AP_ is shown with detailed views of the active site, the iron–sulfur metal center, and the OG recognition site, with the simulated annealing composite omit map contoured at the 1.4‐*σ* level and the anomalous map contoured at the 6‐*σ* level (orange). Note the alternate conformations for the [4Fe‐4S] metal center and *alpha* stereoconfiguration for AP product. Solvent molecules, modelled as waters with unit occupancy and temperature factors ranging from 28–35 Å^2^ were found nearby the AP product and Asp144 with differing peak electron density values as follows: *a*, 1.6 *σ*; *b*, 2.7 *σ*; *c*, 3.4 *σ*.

The structures of *Gs* MutY with replacement of Glu43 presented here are comparable to those previously described for MutY complexed to DNA. The enzyme is crescent shaped with two domains connected by a linker nearly encircling the DNA (Figure 3). Strong electron density defined the replacing residue at position 43 and the DNA at the active site (Figure 3). The NTD inserts intercalating wedge residues Gln48 and Tyr88 into the minor groove to bend the DNA at an angle of ∼50°. The substrate base (Figure 3A,B) or AP product (Figure 3C) is extruded into the active site defined by residues Tyr126, Asp144, and Asn146 along with the replacing residue at position 43. Interactions with the orphaned, yet intra‐helical OG base in these structures were preserved as described for previous structures [12, 20, 30]. Figure 3C shows a representative detailed view of the OG lesion as found in the E43S_OG:AP_ structure. The peptide carbonyl group of Gln48 and the side chain of Thr49 make hydrogen bonds with N^1^ and N^2^ along the Watson–Crick–Franklin face of OG. The CTD contributes the FSH motif at the tip of a *beta* loop [20], with Ser308 making OG‐specific hydrogen bonds with H^7^ and O^8^, atoms that distinguish OG from undamaged guanine. An iron–sulfur cluster [4Fe‐4S] is chelated by four Cys residues in the NTD. For the E43S_OG:AP_ structure captured at relatively high resolution (1.68 Å) two alternate conformations for the [4Fe‐4S] metal center were distinguishable in the electron density maps, especially for the anomalous difference maps (Figure 3C). Alternate conformations for the [4Fe‐4S] metal center have been noted in other high‐resolution views of *Gs* MutY with amino acid replacements [13, 31].

### 2.4 Enzyme Activity by Crystallography

The three structures selected for complete refinement were representative of several crystals with the same substrate versus product status. In other words, we have reproducible results applying X‐ray crystallography to diagnose progress of the enzyme‐catalyzed glycosylase reaction. Outcomes as observed in electron density maps obtained from multiple crystals of each class are summarized in Table S2. Substrate was consistently observed in four crystals for the E43S variant complexed with DNA containing dA across from the OG lesion. Electron density was missing for the base moiety in seven examples for the E43S variant originally complexed with DNA containing dP across from the OG lesion. The three crystals measured for E43Q complexed with the OG:dP lesion consistently revealed density for the base moiety, albeit with weaker signal in |Fo| ‐ |Fc| difference maps, indicating partial conversion to product. The group occupancy for the purine base in the fully refined E43Q_OG:dP_ structure was 0.7. Thus, crystallography corroborates our finding that Glu43 replacement impairs but does not completely ablate glycosylase activity.

The presence of substrate base or AP product in crystals likely reflects multiple processes including enzyme catalysis and crystal growth. Divalent metal ions such as Ca^2+^ included with crystallization reactions inhibit *Gs* MutY and *Gs* MutY (N146S) [13]. To encourage intermediates and products, DNA and enzyme were combined and incubated at ambient temperature for 30–90 min or at 4°C for several hours before adding crystallization solutions with calcium acetate (Table S5). We speculate that slow enzyme catalysis favors crystals with the substrate base and also crystals with the AP product. Rapid catalysis may have prevented growth of crystals, perhaps due to AP product instability. This model explains why the E43Q enzyme, which is faster than E43S (Figure 2), could not be crystallized with DNA containing dA across from OG, the substrate that is processed faster than dP (Figure S1), and why the wild‐type enzyme failed to crystallize with either substrate.

For the E43S enzyme and DNA with the OG:dP lesion, catalysis and crystallization apparently collaborated to generate the enzyme‐bound AP product trapped in crystals. DNA with OG across from dP was a poor substrate in glycosylase assays for E43S and E43Q (Figure S1). With 60% conversion to product by E43S acting on OG:dP during prolonged (24‐h) incubation at 60°C, preincubation of enzyme and DNA for 30 min at ambient temperature would not be sufficient to accumulate significant product from the dP substrate. To explain 100% AP product in the crystals of E43S_OG:AP_, metal ions must have failed to inhibit the E43S variant enzyme so that catalysis proceeded concurrently with and possibly after crystallization. In support of this model, enzyme activity has been demonstrated *in crystallo* for *Gs* MutY (N146S) [13].

### 2.5 Active Site Structure for Substrate

**FIGURE 4:**
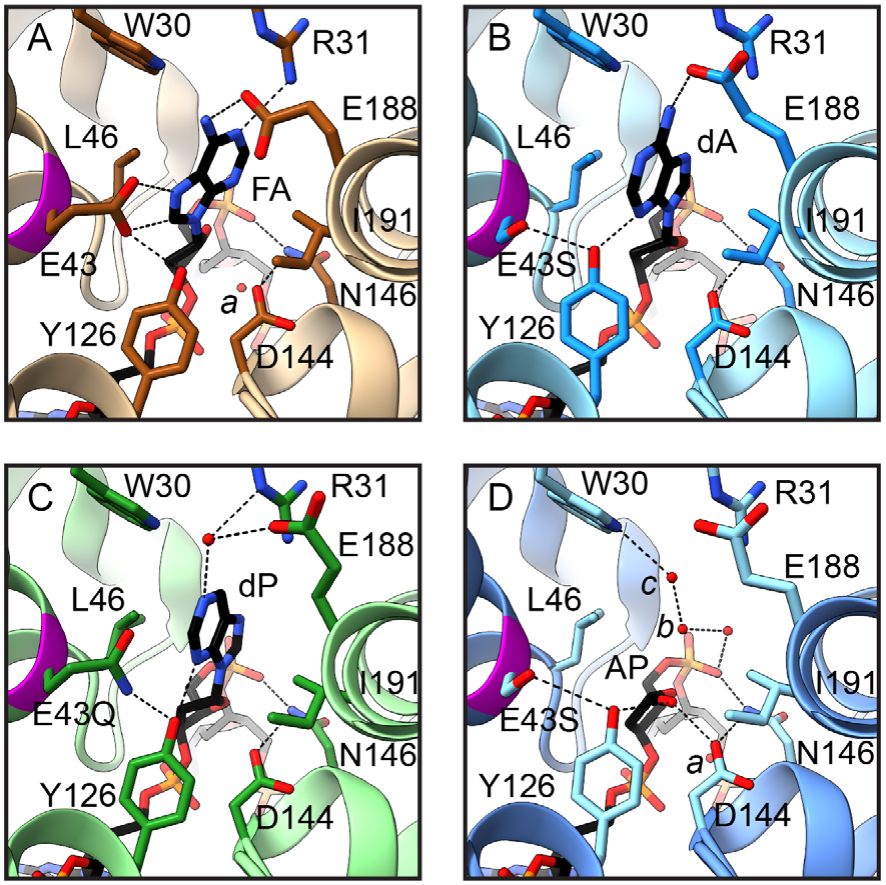
Active site structures. (A) The nucleobase of 2′‐fluoro‐2′‐deoxy‐adenosine (FA) orients in a *syn* conformation (Chi‐1 = −30.5°) and hydrogen bonds with Glu43 and Glu188 in the active site of the FLRC (PDB ID 3g0q). (B) Adenine (dA) orients in an *anti* conformation (Chi‐1 = −172°) and hydrogen bonds with Tyr126 and Glu188 in the active site of the E43S variant enzyme. (C) Purine (dP) orients in the *anti* conformation (Chi‐1 = −186°) and hydrogen bonds with Tyr126 in the active site of the E43Q variant enzyme. For each of these substrate complexes the nucleobase is sandwiched between Leu46 and Ile191. (D) The AP product, generated by E43S acting on DNA with the OG:dP lesion, is observed as the ring‐closed *alpha* anomer. Solvent molecules nearby the AP site are labelled (*a*, *b*, *c*).

To structurally evaluate the contribution of Glu43, we focused attention on the active site of MutY looking for elements that were preserved and things that changed in response to replacement of Glu43. For the Ser substituted variant with adenine in the active site, E43S_OG:A_, we observed tight fitting electron density for a small residue at position 43 (Figure 3A), consistent with Ser replacing Glu. Previous structures of wild‐type *Gs* MutY, complexed with DNA containing a noncleavable substrate analog [11], showed Glu43 interacting with Tyr126 and engaged with N^7^ of the 2′‐fluoro‐2′‐deoxy‐adenine (Figure 4A). Relative to this reference structure, Ser43 shows a substantially different and reduced set of interactions (Figure 4B). Tyr126 has shifted to partly fill the space vacated by Glu43 replacement. Other active site residues preserved interactions seen in previously described structures. For example, catalytic residues Asp144 and Asn146 maintained hydrogen bonding interactions with each other and the DNA backbone. From this we may conclude that perturbations consequent to E43S replacement were fairly localized.

Local perturbations to DNA structure were evident. The adenine base adopted an *anti* orientation (Figures 3A and 4B), with O^4^’ and C^4^ in *trans* and rotated by 180° relative to the *syn* conformation observed for prior substrate complexes, including the lesion recognition complex (LRC) [30], the FLRC [11], and a recently described structure for the N146S variant of *Gs* MutY complexed with purine (N146S_OG:dP_) [13]. Evidence for the unexpected *anti* conformation comes from maps calculated after initial molecular replacement, which outlined a nucleobase with an exocyclic group (Figure S3). Replacement of Glu43 and rotation of the N–glycosidic bond change the hydrogen‐bonding interactions available to the nucleobase. Glu43 of the wild‐type enzyme approaches closely with N^7^ and C^8^ of FA in the *syn* conformation (Figure 4A) [11]. In the *anti* conformation, N^7^ fails to find a partner and N^3^ of dA hydrogen bonds with the hydroxyl group of Tyr126. Glu188 adapts its rotamer configuration to preserve the hydrogen bond with exocyclic N^6^ (Figure 4B). Switching from *syn* to *anti* is coupled with a different sugar pucker: C^2^’‐*endo* for *syn* becomes C^3^’‐*exo* for *anti*. The net effect preserves the position of the nucleobase, sandwiched between Leu46 and Ile191.

We also observed the *anti* conformation with C^3^’‐*exo* sugar pucker for the dP nucleobase in the E43Q_OG:dP_ structure (Figures 3B and 4C), an outcome that indicates the *anti* conformation does not rely on N^6^ interactions that are available for dA but absent for dP. We also infer that, in the absence of Glu43, the *syn* orientation is unstable relative to the *anti* orientation. In the E43Q_OG:dP_ structure, Gln43 hydrogen bonded with Tyr126 and adopted the same rotamer as seen for Glu43, meaning it was in the correct position to interact with a *syn* nucleobase. However, the dP nucleobase remained disengaged from Gln43 by rotation about the N–glycosidic bond. We may conclude that shifting the *anti‐syn* equilibrium toward the *syn* conformation is coupled with the strongly shared proton connecting glutamic acid of the wild‐type enzyme and the adenine nucleobase.

Glutamic acid at position 43 is understood to be the acid/base catalyst that initiates adenine excision by protonation of N^7^ (Figure 1) [11]. We examined the structures looking for other residues that could serve as an alternate acid/base catalyst in the E43S and E43Q variant enzymes. E188 makes a direct hydrogen bond with N^6^ of dA in the structure of E43S_OG:dA_ (Figure 4B). A solvent molecule bridges N^1^ of dP to E188 and R31 in the structure of E43Q_OG:dP_ (Figure 4C). Additionally, Tyr126 hydrogen bonds with N^3^ of the nucleobase in the structures for E43S_OG:dA_ and E43Q_OG:dP_ (Figure 4B,C). Findings from SAR analyses support N^3^ involvement for adenine excision. The wild‐type enzyme from *E. coli* (*Ec* MutY) excised a hydrophobic isostere of adenine that lacked N^7^ and retained N^3^, 7‐deaza‐adenine [32]. Additionally, the isostere lacking N^3^, 3‐deaza‐adenine, was excised at a rate 150‐fold slower relative to the authentic substrate [16]. It is therefore reasonable to consider proton transfer from Tyr126 to N^3^ as a plausible first step for adenine excision by the impaired E43S and E43Q enzymes (Figure S5).

### 2.6 Structure for the Enzyme‐Generated Product

Electron density for a nucleobase was missing in the E43S_OG:AP_ structure. Absence of electron density for the purine base in the discovery maps calculated for initial rounds of refinement, well defined simulated annealing composite omit maps (Figure 3C), and difference maps calculated for the final model refined with data to the 1.68 Å resolution limit (Figure S4B) provide strong evidence that the base has been excised. The detached free base was evidently not retained following cleavage of the N–glycosidic bond. Attempts to build a nucleobase into electron density features nearby the sugar group yielded unsatisfactory results. These features were modeled as solvent molecules, specifically water with unit occupancy which refined with reasonable temperature factors (Figure 3C). Electron density for the abasic site defined an AP group in its closed‐ring furanose form, with density extending beyond C^1^’ in an equatorial disposition, consistent with a hydroxyl group in the *alpha* stereoconfiguration (Figures 3C and S4B) and very different from the *beta* stereoconfiguration observed for the AP product generated by *Gs* MutY N146S (Figure S4A) [13].

We took special care in selecting the C^4^’‐*endo* sugar pucker and *alpha* stereoisomer for the AP site. Sugar conformations that avoid an axial disposition for the O^1^’ atom were compatible with electron density maps, including C^4^’‐*endo*, C^2^’‐*endo* and C^1^’‐*exo*, each with *alpha* configuration at C^1^’ (Figures 3C and S8). The *alpha* anomer with C^4^’‐*endo* pucker was selected because it fit electron density well with minimal features in difference maps, refined with reasonable temperature factors for all atoms in the AP group, and avoided steric clashes (Table S7).

The *alpha* anomer places the O^1^’ group within hydrogen bonding distance of Asp144 and Tyr126 (Figure 4D) and was unexpected since previous studies show that wild‐type *Gs* MutY with Glu converts substrate to the *beta* anomer in both methanolysis and hydrolysis reactions [12, 13]. Comparison of the E43S_OG:AP_ structure described here with N146S_OG:AP_, described previously [13], indicates Glu43 replacement has dramatically altered interactions with the AP product (Figure S4), and suggests an alternative inverting mechanism contributes to product accumulation. The alternative mechanism presented in Figure S5 is one of several possibilities that lead to the AP *alpha* anomer for the impaired enzyme with replacement at position 43. For instance, we speculate that the nucleobase is in its *anti* conformation prior to excision. An alternate scenario involving *anti‐syn* switching prior to excision may also contribute. However, the tight packing of groups around the nucleobase imposes strong barriers to *anti‐syn* switching, which is even less likely in the crystalline state where opportunities for DNA dissociation and rebinding are limited.

### 2.7 Implications for MutY's Catalytic Mechanism and Regulation

Our work reinforces and extends the idea that recruitment of a catalytic glutamic acid residue was a cornerstone innovation for the evolution of an OG:A‐specific adenine DNA glycosylase with a retaining mechanism. DNA glycosylases are tasked with finding and precisely identifying rare substrates among vast amounts of normal DNA, placing evolutionary pressure to finetune substrate specificity. The search for substrate while avoiding off‐target decoys is made especially challenging for MutY, which excises an undamaged adenine nucleobase. Most other glycosylases remove a damaged base, and therefore can take advantage of distinct chemical groups for recognition and the inherent instability of the N–glycosidic bond following chemical modification [33, 34, 35, 36]. In the absence of chemical damage, excision of the adenine base places additional demands for substrate recognition.

Since the nucleobase being excised is highly prevalent, MutY must strictly integrate information from the OG‐recognition site to distinguish appropriate OG:A substrates. Off target activity would lead to massive AP accumulation and genome instability. This molecular logic makes clear the need for an “*on hold*” state that precedes the fully licensed catalytic state of MutY. Release of the “*on hold*” state likely involves allosteric communication among distant sites, including the OG‐site, the active site, and the [4Fe‐4S] metal center. Connectivity with the metal center is supported by recently reported new structures for *Gs* MutY and human MUTYH complemented by molecular dynamic analysis [31]. In that work, variants of MutY that interrupt the connection to the [4Fe‐4S] metal center retain structure, bind substrate DNA normally, yet are catalytically disabled [31]. Recent large‐scale enhanced‐sampling simulations highlight structural transitions involving *anti/syn* switching for both OG and A nucleobases to progress from the encounter complex to the catalytically engaged LRC [37]. Failure to accomplish these transitions would prevent capture of the A nucleobase in its *syn* orientation by catalytic residues in the active site.

The X‐ray crystal structures we present here illustrate that a nucleobase captured in its *anti* orientation cannot make productive interactions with catalytic residues. With the adenine nucleobase in an *anti* conformation, Glu43 would be disengaged from N^7^, thereby keeping catalysis in check. In this model, switching to a *syn* conformation is necessary to establish the “*go ahead*” state characterized by catalytic engagement between the glutamic acid and the nucleobase for efficient adenine excision. This logic applies to any errant nucleobase extruded from the DNA helix. If these enter the active site accidentally, they will likely do so in the default *anti* orientation and thereby fail the test for an authentic substrate. Only those adenine nucleobases paired with OG pass the test by entering the active site in their *syn* conformation.

### 2.8 The Covalent Intermediate

The retaining mechanism of MutY predicts a covalent DNA‐enzyme intermediate [12, 13], and a covalent crosslink from C^1^’ in DNA to the carboxylate of Asp144 in MutY has been observed in QM/MM structures [15]. The hope of capturing this covalent DNA‐MutY intermediate in a crystal structure provided impetus for exploring Glu43 replacement variants. We reasoned that purine, with its enhanced leaving group potential, would increase the rate of forming the covalent intermediate, while Glu replacement would slow its destruction. Replacement of Glu with Gln led to capture of covalent enzyme‐glycosyl intermediates for hen egg white lysozyme and the human nucleotide salvage factor DNPH1 [38, 39]. However, expectations were not fulfilled for our MutY system. Substrate purine was processed by E43S and E43Q enzymes only very slowly (Figure S1), suggesting that purine is a poor leaving group for the enzyme‐catalyzed reaction, perhaps consequent to adopting the *anti* orientation. The “*on hold*” state proposed for regulating MutY catalysis may further explain why the expected covalent intermediate of MutY eluded capture. Release of the “*on hold*” state necessitates kinetic steps preceding catalysis. If any of these steps are slow, then the MutY covalent intermediate will remain poorly populated.

### 2.9 Comparison of Catalytic Strategies for Adenine Glycosylases

Comparison of MutY's catalytic strategy with those for other adenine glycosylases suggests different constraints for their evolution. MutY, the ribosome inactivating proteins (RIPs) from plants, and the purine‐specific nucleoside hydrolase (NH) from *Trypanosoma vivax* each solve the challenge of breaking a *beta*‐N–glycoside bond connecting C^1^’ to N^9^ of adenine. As these proteins have completely different folds, the comparable functions represent convergent evolution. KIE analyses indicate dissociative SN1 mechanisms for these enzymes, with differences in transition state structure [14, 40, 41, 42].

One residue serves multiple catalytic functions for the MutY mechanism. The catalytic Glu is the acid/base catalyst for activation of the leaving group, activation of the nucleophile, and contributes, along with the catalytic Asp (Asp144), to electrostatic stabilization of the positively charged transition states encountered before and after the covalent intermediate (Figure 1). For RIPs and purine‐specific NH, these catalytic tasks are delegated to several residues. The RIP saporin‐L3 accomplishes leaving group stabilization by an extended *π*‐bond stacking interaction involving an Arg and two Tyr residues coupled with protonation at N^3^ by a conserved and catalytically critical Arg [43]. These residues devoted to leaving group activation are different from those that provide electrostatic stabilization of the oxacarbenium ion transition state and nucleophile activation, tasks fulfilled by a catalytic Glu [44]. Comparable to RIPs, the purine‐specific NH also stabilizes the leaving group via *π*‐bond stacking interactions with two Trp residues [45, 46].

**FIGURE 5:**
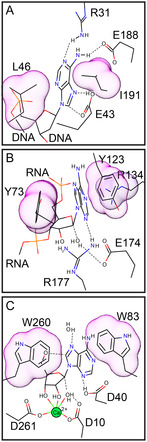
Comparison of DNA, RNA, and nucleoside adenine glycosylases. Adenine‐sandwiching residues are highlighted. (A) MutY excises adenine from DNA and sandwiches the nucleobase with nonaromatic, aliphatic residues. (B) Saporin‐L3 excises adenine from RNA and sandwiches the leaving group with two aromatic Tyr residues, supported by an Arg. (C) Purine specific nucleoside hydrolase sandwiches the nucleobase leaving group with two aromatic Trp residues. Drawn on the basis of PDB IDs 3g0q [11], 2ff2 [43], and 3hiw [45].

The stacking interactions featured in leaving group activation for RIPs and NH are absent for MutY (Figure 5). Residues sandwiching the adenine nucleobase in MutY are hydrophobic, aliphatic residues, and aromatic residues are avoided at these positions among orthologs from bacteria (Figure S6). Inspection of amino acid sequence alignments shows Phe is accepted at these adenine‐sandwiching positions at a frequency of ∼1%, indicating aromatic residues may be accommodated. There are no examples with both positions occupied by an aromatic residue. Avoidance of aromatic residues makes nucleobase activation by MutY completely reliant on engagement with the catalytic Glu and may serve to prevent premature, inappropriate adenine excision by MutY.

Different biological purposes likely contributed to differences in catalytic strategy. Efficient turnover realized for RIPs reflects their evolved purpose to disable numerous ribosomes in a cell as quickly as possible. The biological purpose of nucleotide hydrolases varies, but a high turnover was apparently desirable. By comparison, MutY is marked by slow turnover, a result of strong product inhibition [6]. High catalytic turnover is not a priority for MutY as there are few authentic substrates in the cell. Instead, evolution invested in an ultra‐efficient search for rare adenine substrates paired with OG [24]. The proposed “*on hold*” state would provide protection to ensure MutY serves a DNA‐repair function even as it breaks the N–glycosidic bond at chemically correct but informationally misplaced adenines. We suggest that establishing and releasing such a checkpoint is facilitated by strong reliance on one residue, the catalytic Glu, for leaving group activation.

## 3 Conclusion

**FIGURE 6:**
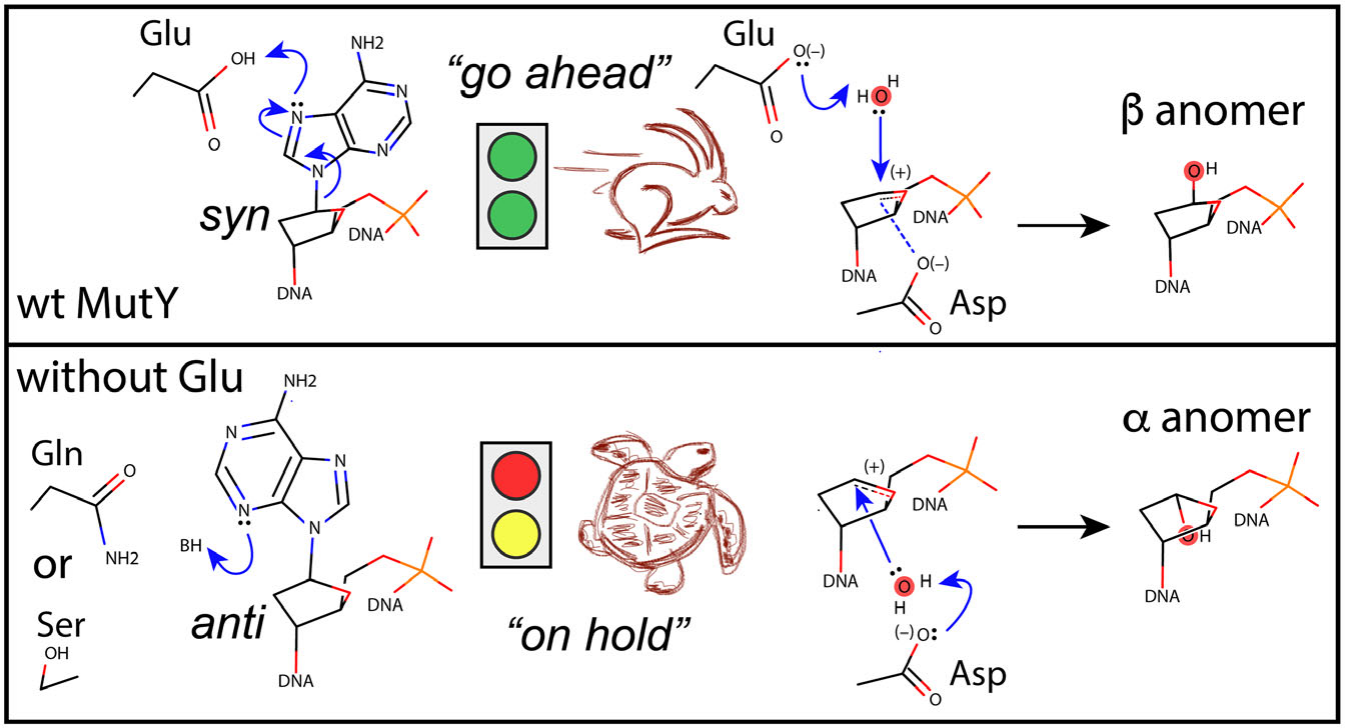
The importance of Glu for MutY. The catalytic Glu activates both the leaving group in a *syn* conformation and the nucleophile for efficient adenine excision with retention of stereochemistry (top). As revealed in this article describing structures and kinetics for E43S and E43Q replacement variants of *Gs* MutY, in the absence of Glu the nucleobase adopts an *anti* conformation, the reaction is very slow, and the resulting AP product is observed in a furanose ring‐closed form with *alpha* stereoconfiguration. These features contribute to our understanding of MutY's function to restore DNA in the face of oxidative damage.

The outcomes described here are summarized in Figure 6. Replacement of the catalytic Glu with Ser or Gln reduced the adenine excision rate by four orders of magnitude. Structures of these Glu43 replacement variants in complex with DNA showed the substrate nucleobase in an unfamiliar *anti* orientation and the enzyme‐generated AP product in a closed‐ring, *alpha* anomer configuration, very different from the *beta* stereoisomer seen for the N146S variant enzyme in crystal structures [13], and as inferred for the wild‐type enzyme on the basis of enzyme‐catalyzed methanolysis [12]. We knew from previous work that Glu is critical for facilitating initial steps involving acid/base catalysis to activate the leaving group [14, 29]. The results described here are consistent with the same catalytic Glu also activating the nucleophile to attack from the *beta* face, the same face as leaving group departure. Other adenine excising enzymes acting on RNA and nucleosides distribute catalytic tasks for nucleobase and nucleophile activation among many residues to establish an SN1 inverting mechanism, while MutY relies heavily on the one catalytic Glu for both nucleobase and nucleophile activation in an SN1 retaining mechanism. For MutY, an “*on hold*” state, marked by Glu disengagement and released only in response to OG recognition *via* an allosteric network, would allow for licensed DNA restoring function at rare adenines paired with oxidized guanine.

## 4 Experimental Section

See Supplementary Information for the Experimental Section.

## Funding

This study was supported by National Science Foundation (Grant CHE‐2204228 and CHE‐2204229).

## Conflicts of Interest

The authors declare no conflicts of interest.

## Supporting information

 The authors have cited additional references within the Supplementary Information [6, 11, 12, 13, 20, 23, 32, 49, 50, 51, 52, 53, 54, 55, 56, 57, 58, 59, 60, 61, 62, 63, 64].

## Data Availability

Plasmid DNAs have been archived with AddGene with ID numbers 254 899 and 254 900. Protein structures have been deposited with the Protein Data Bank [47, 48], with PDB IDs 8dwd, 8dwe and 8dwf.
